# Effects of melatonin on cardiovascular diseases: progress in the past year

**DOI:** 10.1097/MOL.0000000000000314

**Published:** 2016-04-25

**Authors:** Hang Sun, Aaron M. Gusdon, Shen Qu

**Affiliations:** a Department of Endocrinology and Metabolism, Shanghai Tenth People's Hospital, School of Medicine, Tongji University, Shanghai, China; b Department of Pathology, Division of Neuropathology, University of Pittsburgh School of Medicine, Pittsburgh, Pennsylvania; Department of Neurology, Weill Cornell Medical College, New York, USA

**Keywords:** cardiovascular diseases, melatonin, myocardial ischemia-reperfusion injury

## Abstract

**Purpose of review:**

Melatonin is a neuroendocrine hormone synthesized primarily by the pineal gland. Numerous studies have suggested that melatonin plays an important role in various cardiovascular diseases. In this article, recent progress regarding melatonin's effects on cardiovascular diseases is reviewed.

**Recent findings:**

In the past year, studies have focused on the mechanism of protection of melatonin on cardiovascular diseases, including myocardial ischemia-reperfusion injury, myocardial hypoxia-reoxygenation injury, pulmonary hypertension, hypertension, atherosclerosis, valvular heart diseases, and other cardiovascular diseases.

**Summary:**

Studies have demonstrated that melatonin has significant effects on ischemia-reperfusion injury, myocardial chronic intermittent hypoxia injury, pulmonary hypertension, hypertension, valvular heart diseases, vascular diseases, and lipid metabolism. As an inexpensive and well tolerated drug, melatonin may be a new therapeutic option for cardiovascular disease.

## INTRODUCTION

Melatonin (N-acetyl-5-methoxytryptamine) is a neuroendocrine hormone, which is synthesized primarily by the pineal gland [1]. The synthesis and secretion of melatonin are regulated by light intensity [2]. It was found that melatonin functions to regulate the sleep cycle in the early study [3]. Further investigation revealed that melatonin also has antioxidant and anti-inflammatory functions [4]. It has also been shown to regulate lipid and glucose metabolism [5,6]. Importantly, recent research suggests that melatonin plays an important role in various cardiovascular diseases, including myocardial ischemia-reperfusion injury [7,8], atherosclerosis [9,10], hypertension [11,12], heart failure [13,14], and drug-induced myocardial injury [15,16]. In the past year, several studies have focused on the mechanism of the protection of melatonin on cardiovascular diseases. In this article, we review the recent progress in the understanding of melatonin's effects on cardiovascular disease. 

**Box 1:**
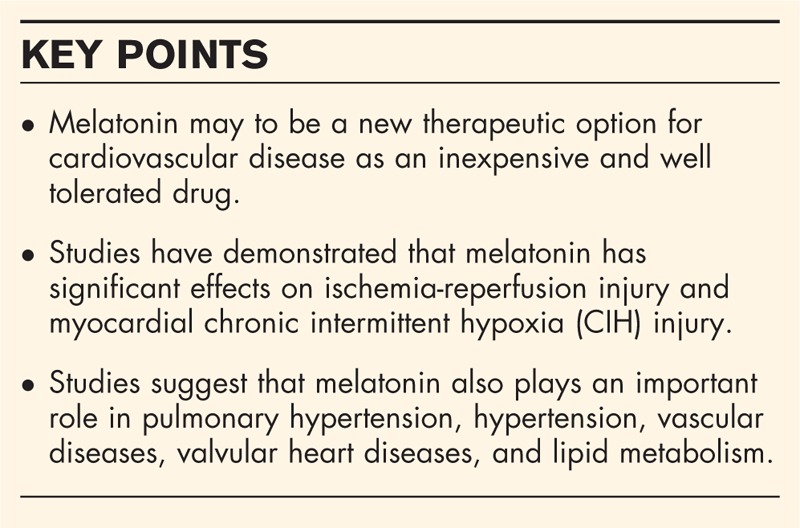
no caption available

## MELATONIN AND MYOCARDIAL ISCHEMIA-REPERFUSION INJURY

Melatonin confers profound protective effects against ischemia-reperfusion injury in various organs, including the heart [7,8], liver [17], and kidney [18]. However, the mechanisms by which it affords protection remain incompletely understood. Ghaeli *et al.*[19] reported that in patients with ST-segment elevation myocardial infarction undergoing primary percutaneous coronary intervention, administration of melatonin plus standard treatment significantly reduced the level of creatine kinase-MB compared with the control group, receiving only standard therapy. However, in a porcine closed-chest reperfusion infarct model, intracoronary or intravenous melatonin administration did not reduce myocardial reperfusion injury [20]. The lack of effect may be because of the ineffective dose and route of administration. It was found that melatonin may protect against ischemia-reperfusion injury by activating silent information regulator 1 (SIRT1) signaling in a receptor-dependent manner [21]. Another study found that melatonin could protect adipose-derived mesenchymal stem cells (ADSCs) against hypoxia/serum deprivation injury by modulating the SIRT1 signaling pathway. Melatonin treatment also reduced the expression of the apoptotic proteins acetylated forkhead box protein O1 (Ac-Fox01), acetylated p53 (Ac-p53), acetylated nuclear factor kappa-light-chain-enhancer of activated B cells (Ac-NF-kB), and B-cell lymphoma 2 (BCL2)-associated X protein (BAX), while increasing the expression of the antiapoptotic protein B-cell lymphoma 2 (BCL-2) [22^▪^]. Similarly, melatonin improved the survival and function of ADSCs in a rat model of myocardial infarction [23]. Its protective effects were due to increased expression of Cu/Zn superoxide dismutase (SOD-1) and other antioxidant enzymes, basic fibroblast growth factor, insulin-like growth factor 1, epidermal growth factor, and hepatocyte growth factor. Melatonin also protected mesenchymal stem cells (MSCs) against reactive oxygen species (ROS)-induced apoptosis by stimulating phosphorylated protein kinase B (p-Akt) and inhibiting activation of the caspase cascade [23]. Yu *et al.*[24] studied the effect and mechanism of melatonin on myocardial ischemia-reperfusion (MI/R) by modulating Notch1/Hairy and enhancer of split 1 (Hes1) and phosphatase and tensin homolog (PTEN)/Akt signaling pathways. In an in-vivo study, prophylactic use of melatonin before myocardial ischemia-reperfusion surgery significantly improved cardiac function, reduced oxidative damage, and decreased myocardial apoptosis [24]. In an in-vitro study of H9C2 cardiomyocytes, melatonin treatment increased Notch1, Notch1 intracellular domain, BCL-2, and Hes1 expression and the ratio of phosphorylated to unphosphorylated Akt, while reducing caspase-3, PTEN, and BAX expression [24]. Another study showed that melatonin significantly inhibited myocardial apoptosis during myocardial ischemia-reperfusion in rats [25]. Melatonin also preserves the structural integrity of mitochondria in myocardiocytes, promoting adenosine triphosphate synthesis and preserving cardiac function [25]. In a rat model of ischemia/reperfusion injury, administration of melatonin reduced infarct size by inhibiting the mitochondrial permeability transition pore [26]. In a model of diet-induced obesity utilizing Wistar rats, melatonin treatment reduced serum insulin levels, homeostatic model assessment index and myocardial infarct volume, while increasing serum adiponectin levels and activating baseline myocardial extracellular signal-regulated kinases 42/44 (ERK 42/44), glycogen synthase kinase-3 beta (GSK-3 β), signal transducer and activator of transcription 3 (STAT-3), and Protein Kinase B (PKB/Akt) during reperfusion [27]. In another rat model of high-fat diet-fed streptozotocin induced diabetes [28^▪^], treatment with melatonin suppressed protein kinase ribonucleic acid-like endoplasmic reticulum kinase (PERK)/eukaryotic initiation factor 2 alpha kinase/activating transcription factor 4 (ATF4) signaling, reduced myocardial oxidative damage, and up-regulated SIRT1 expression. Endoplasmic reticulum stress is considered to be an important contributing factor in cardiovascular diseases [29]. Melatonin was also found to modulate endoplasmic reticulum stress by suppressing PERK/eIF2α/ATF4 signaling after ischemia reperfusion in H9C2 cardiomyocytes [28^▪^]. A recent study also implicated Toll-like receptor 4 (TLR4) signaling in the protective effects of melatonin [30^▪^]. In isolated hearts, melatonin treatment was shown to protect against ischemia/reperfusion injury through increased TLR4 signaling and also increased mitochondrial STAT-3 expression, leading to subsequent activation of the survivor activating enhancement pathway [30^▪^].

## MELATONIN AND MYOCARDIAL CHRONIC INTERMITTENT HYPOXIC INJURY

Obstructive sleep apnea is associated with CIH and increases myocardial injury contributing to ischemic heart disease [31]. Yeung *et al.*[32^▪^] reported that melatonin protected against CIH-induced myocardial inflammation, fibrosis, and ischemia-reperfusion injury. In this study, treatment with melatonin significantly reduced the expression of inflammatory cytokines [tumor necrosis factor-α (TNF-α) and IL-6] and markers of fibrosis [PC1 and transforming growth factor β (TGFβ)]. Furthermore, melatonin treatment decreased infarct size in isolated hearts with regional ischemia reperfusion by mitigating sarcoplasmic reticulum calcium (2+) [SR-Ca(2+)] homeostasis in cardiomyocytes and reducing the expression of nicotinamide adenine dinucleotide phosphate oxidase (p22 and NOX2) and antioxidant enzymes [catalase (CAT) and manganese-superoxide dismutase (MnSOD)]. Xie *et al.*[33] reported that melatonin decreased CIH-induced myocardial hypertrophy and cardiomyocyte apoptosis by modulating the adenosine monophosphate-activated protein kinase pathway and autophagy-regulated apoptosis. Using a melatonin receptor agonist, Neu-p11, Yu *et al.*[34] demonstrated that downstream signaling protected myocardial cells from hypoxia-reoxygenation injury. Administration of Neu-p11 reduced cardiomyocyte apoptosis and also significantly decreased levels of creatine kinase, lactate dehydrogenase (LDH), and methane dicarboxylic aldehyde. Cardiovascular diseases have been associated with mitochondrial dysfunction [35]. Importantly, Neu-p11 also protected mitochondria from ischemia-reperfusion-mediated injury and modulated lipid peroxidation. Ortiz *et al.*[36] found that melatonin treatment inhibited iNOS/i-mtNOS (iNOS, inducible nitric oxide synthase; i-mtNOS, inducible mitochondrial nitric oxide synthase) induction, restored myocardial mitochondrial homeostasis and preserved the activity of nNOS/c-mtNOS (nNOS, neuronal nitric oxide synthase; c-mtNOS, constitutive mitochondrial nitric oxide synthase). Additionally, a study using chick embryos showed that melatonin (1 mg/kg/day) rescued hypoxia-induced cardiovascular dysfunction [37].

## MELATONIN AND PULMONARY HYPERTENSION

Pulmonary hypertension is a disease characterized by elevated pulmonary arterial pressure, which leads to right ventricular hypertrophy and failure [38]. Maarman *et al.*[39] reported that treatment with melatonin alleviated right ventricular hypertrophy and dysfunction, and also reduced interstitial fibrosis and plasma oxidative stress in a rat model of pulmonary hypertension. Torres *et al.*[40] found that melatonin reduced pulmonary artery pressure and resistance and improved vasodilation of small pulmonary arteries in newborn sheep with pulmonary hypertension. In addition, melatonin increased nitric oxide bioavailability and reduced markers of pulmonary oxidative stress. Jin *et al.*[41^▪^] reported that melatonin attenuated hypoxic pulmonary hypertension. Chronic hypoxia elevates the ratio of the weights of the right ventricle to left ventricle plus intraventricular septum (RV/LV+S), right ventricular systolic pressures (RVSP), and median width of pulmonary arterioles. Treatment with melatonin reduced the elevation of RV/LV+S and RVSP and also inhibited pulmonary vascular remodeling. Additionally, melatonin reduced levels of hypoxia-inducible factor-1α, proliferating cell nuclear antigen, and nuclear factor-κB (NF-κB). In an in-vitro study, it was found that melatonin inhibited the proliferation of pulmonary artery smooth myocytes and reduced the expression of extracellular signal-regulated kinases1/2 (ERK1/2) and phosphorylation of Akt.

## MELATONIN AND HYPERTENSION

Several studies have demonstrated that melatonin has an antihypertensive effect [42,43]. Simko *et al.*[44] found that melatonin alleviated hypertension is induced by continuous light exposure (24 h/day). Continuous light leads to hypertension, increased oxidative stress in the left ventricle and aorta, left ventricle hypertrophy, and left ventricle fibrosis. Melatonin treatment alleviated these pathological changes. İlhan *et al.*[45] also reported that melatonin alleviated 2,3,7,8-tetrachlorodibenzo-p-dioxin-induced hypertension by decreasing renal oxidative stress and vascular reactivity. A recently published review has confirmed the blood pressure (BP) lowering effects of melatonin [46]. It was shown that patients treated with melatonin (2–5 mg/day for 7–90 days) had a decrease in nocturnal SBP as well as DBP [46]. Additionally, it was demonstrated that the effect of melatonin on decreasing BP were most pronounced from 3:00 am to 8:00 am [47].

## MELATONIN AND VASCULAR DISEASES

Recent studies have shown that melatonin is associated with atherosclerosis [48,49]. Cheng *et al.*[50] reported that melatonin reduced the number and area of atheromatous plaques in a rabbit model of atherosclerosis by modulating mitogen-activated protein kinase (MAPK) pathway signal transduction. In addition to MAPK signaling, a recent study showed that melatonin decreased aortic endothelial permeability and atherosclerosis in a mouse model of diabetes by decreasing the expression of myosin light chain kinase (MLCK), myosin phosphatase-targeting subunit phosphorylation, and myosin light-chain phosphorylation. Melatonin also decreased upstream expression of extracellular signal-related kinase (ERK) and p38 [51]. Zhu *et al.*[52] found that micro ribonucleic acid-29b (miR-29b) promotes endothelial permeability and apoptosis in high-fat diet-fed apoE knock-out mice by down-regulating the expression of MT1, which is a melatonin receptor. Yang *et al.*[53] reported that the anti-inflammatory effects of melatonin improved cigarette smoke-induced restenosis in rat carotid arteries after balloon injury. Melatonin may improve vascular dysfunction by affecting epigenetic regulation. In mice generated with assisted reproductive technologies, treatment with melatonin resulted in decreased arterial hypertension, which was thought to be due to its effects on normalizing nitric oxide levels by preventing impaired methylation of endothelial nitric oxide synthase [54]. It was also shown that melatonin may improve macrovascular and microvascular diseases [55–58]. Melatonin administration to high-fat diet and streptozotocin-induced diabetic rats restored endothelial function and vascular responses [59].

## MELATONIN AND VALVULAR HEART DISEASE

It has been demonstrated that melatonin reduces flow shear stress-induced bone marrow mesenchymal stem cells injury by acting on melatonin receptors and the adenosine monophosphate-activated protein kinase/acetyl-CoA carboxylase signaling pathway [60^▪^]. In this study, melatonin reduced the expression of caspase 3, p53 upregulated modulator of apoptosis, and BAX, while inducing the expression of basic fibroblast growth factor, TGFβ, vascular endothelial growth factor, (BCL-2), and platelet-derived growth factor [60^▪^]. These findings suggest that targeting melatonin relating signaling in tissue-engineered heart valves may be an effective strategy in treating valvular heart disease.

## MELATONIN AND LIPID METABOLISM

Early experiments showed that treatment with melatonin can improve dyslipidemia [4]. In patients with nonalcoholic fatty liver disease, treatment with melatonin (2 × 5 mg/day) for 14 months significantly reduced levels of triglycerides and LDL cholesterol (LDL-C) compared with controls treated with Essentiale [61]. Treatment with melatonin for 2 weeks significantly reduced free fatty acids compared with placebo in cigarette smokers [62]. A study on aluminum-induced toxicity in a rat model found that melatonin protected against toxic dyslipidemia by alleviating the aluminum induced increase in total cholesterol, LDL-C, triglycerides, oxidized LDL and apolipoprotein B100 [63]. In unpublished results, we have demonstrated that melatonin administration can improve lipid metabolism and reduce weight. Melatonin treatment reduced body weight, body fat, and waist circumference in obese patients with acanthosis nigricans. We also found that melatonin could decrease LDL and body weight in high-fat diet-induced nonalcoholic fatty liver disease mice. Dyslipidemia is an important risk factor of cardiovascular diseases [64], and melatonin's beneficial effects on lipid metabolism may reduce the incidence of cardiovascular diseases.

## CONCLUSION

In conclusion, studies have demonstrated that melatonin has significant effects on ischemia-reperfusion injury, myocardial CIH injury, pulmonary hypertension, hypertension, vascular diseases, valvular heart diseases, and lipid metabolism (Table 1). As an inexpensive and well tolerated drug, melatonin may be a new therapeutic option for cardiovascular disease.

## Acknowledgements

*H.S. wrote the manuscript, A.M.G. and S.Q. were involved in editing the manuscript*.

### Financial support and sponsorship

*None*.

### Conflicts of interest

There are no conflicts of interest.

## REFERENCES AND RECOMMENDED READING

Papers of particular interest, published within the annual period of review, have been highlighted as:

▪ of special interest
▪▪ of outstanding interest

## Figures and Tables

**Table 1:** The effects of melatonin on various cardiovascular diseases

Function	Factor/pathway/action	References
Melatonin and myocardial ischemia-reperfusion injury		
Induce/activate	SIRT1	[21,22^▪^,28^▪^]
	NOTCH1, NICD, HES1, p-Akt/Akt ratio	[24]
	BCL-2,	[22^▪^,^24^]
	SOD-1, HFG	[23]
	Adiponectin, ERK42/44, GSK-3β, STAT-3, PKB/Akt	[27]
	TLR4, STAT3, SAFE	[30^▪^]
Reduce/inhibit	CK-MB	[19]
	BAX	[21,22^▪^]
	Ac-FoxO1, Ac-p53, Ac-NF-κB	[22^▪^]
	Caspase, ROS	[23]
	Caspase-3, PTEN,	[24]
	Serum insulin, HOMA index	[27]
	PERK/eIF2α/ATF4 signaling pathway	[28^▪^]
Melatonin and myocardial chronic intermittent hypoxia injury		
Reduce/inhibit	TNF-α, IL-6, COX-2; PC1, TGF-β; P22, NOX2; CAT, MnSOD	[32^▪^]
	CK, LDH, MDA	[35]
	iNOS/i-mtNOS, nNOS/c-mtNOS	[36]
Melatonin and pulmonary hypertension		
Induce/activate	Nitric oxide	[41^▪^]
Reduce/inhibit	RV hypertrophy and dysfunction, interstitial fibrosis	[39]
	Pulmonary artery pressure and resistance	[40]
	RV/LV+S, RVSP, HIF-1α, PCNA, NF-κb, ERK1/2, p-Akt	[41^▪^]
Melatonin and hypertension		
Reduce/inhibit	Oxidative load in the LV, aorta and LV hypertrophy, LV fibrosis	[44]
	Renal oxidative stress and vascular reactivity	[45]
Melatonin and vascular diseases		
Reduce/inhibit	Number and areas of atheromatous plaques	[50]
	MLCK, p-MYPT, p-MLC, ERK, p-38	[51]
	eNOS	[54]
Melatonin and valvular heart disease		
Induce/activate	bFGF, BCL-2, PDGF	[60^▪^]
Reduce/inhibit	Caspase 3, PUMA, BAX	[60^▪^]
Melatonin and lipid metabolism		
Reduce/inhibit	TG, LDL-C	[61,63]
	FFA	[62]
	TC, oxidized LDL-C, apoB100	[63]

Ac-FoxO1, acetylated forkhead box protein O1; Ac-p53,acetylated p53; Ac-NF-κB, acetylated nuclear factor kappa-light-chain-enhancer of activated B cells; ATF4, activating transcription factor 4; BCL-2, B-cell lymphoma 2; CAT, catalase; ERK42/44, extracellular signal-regulated kinases 42/44; GSK-3 β, glycogen synthase kinase-3 beta; LDH, lactate dehydrogenase; MnSOD, manganese-superoxide dismutase; p-Akt, phosphorylated protein kinase B; PKB, Protein Kinase B; PERK, protein kinase ribonucleic acid-like endoplasmic reticulum kinase; SIRT1, silent information regulator 1; TNF-α: tumor necrosis factor-α; bFGF, basic fibroblast growth factor; CK-MB, creatine kinase-MB; HIF-1α, hypoxia-inducible factor-1α; NICD, NOTCH1 intracellular domain; PCNA, proliferating cell nuclear antigen; PUMA, p53 upregulated modulator of apoptosis; PDGF, platelet-derived growth factor; RV/LV+S, right ventricle to left ventricle plus intraventricular septum; ROS, reactive oxygen species; SOD-1, superoxide dismutase; STAT-3, signal transducer and activator of transcription 3; TLR4, toll-like receptor 4; TGF-β transforming growth factor β; TG, triglycerides. A summary of the mechanistic effects of melatonin on myocardial ischemia-reperfusion injury, myocardial hypoxia-reoxygenation injury, pulmonary hypertension, hypertension, and vascular diseases. (original).
